# Gut microbiota of patients with post-stroke depression in Chinese population: a systematic review and meta-analysis

**DOI:** 10.3389/fcimb.2025.1444793

**Published:** 2025-05-01

**Authors:** Qiaoling Li, Yuejuan Zhang, Xiaoqian Wang, Lin Dai, Wenli Zhao

**Affiliations:** ^1^ Graduate School, Hunan University of Chinese Medicine, Changsha, China; ^2^ Department of Nursing, The First Affiliated Hospital of Hunan University of Chinese Medicine, Changsha, China

**Keywords:** gut microbiome, dysbiosis, PSD, stroke, depression, review

## Abstract

**Background:**

Evidence of changes in the composition and function of the gut microbiota (GM) in post-stroke depression (PSD) patients is gradually accumulating. This study aimed to systematically evaluate the relationship between PSD and GM.

**Methods:**

We searched in PubMed, Web of Science, Embase, Cochrane databases, Wangfang, VIP, CBM, and CNKI from the establishment of the database to April 17, 2024, and systematic review and meta-analysis were performed to investigate the differences of GM between patients with PSD spectrum and healthy controls (HC) or stroke spectrum.

**Result:**

There were 14 studies consisting a total of 1,556 individuals included in the meta-analysis. The pooled results showed that PSD spectrum demonstrated significantly increased α diversity as indexed by Chao1 index, ACE indexes, Shannon index, and Simpson index as compared to HC. Additionally, stroke spectrum significantly increased α diversity as indexed by Simpson index compared to PSD. Furthermore, the pooled estimation of relative abundance showed that *Bacteroidota*, *Fusobacteriota*, and *Pseudomonadota* in PSD patients were significantly higher than those in the HC group, while the abundance of *Bacillota* was higher in the HC group. Moreover, significant differences in GM were observed between PSD patients and HC at the family and genus levels.

**Conclusion:**

This study found that the α diversity of PSD patients was higher than that of HC. Moreover, there were also differences in the distribution of GM at the phylum, family, and genus levels, respectively. At the same time, the level of *Lachnospira* in PSD patients was lower than that in the stroke group.

**Systematic review registration:**

https://www.crd.york.ac.uk/PROSPERO/, identifier CRD42024582708.

## Introduction

Depression and cerebrovascular accident (stroke) are two major causes of socioeconomic burden (Medeiros et al., 2020). The latest report from the World Health Organization (WHO) states that depression is the leading cause of disability worldwide (WHO, 2018a), while stroke often ranks among the top three causes of disease burden (WHO, 2012; WHO, 2018b). The sequelae caused by tissue damage in stroke patients are not easily alleviated over time (Becker, 2016), leading to a variety of lifelong sequelae (Cha et al., 2022), including sensory impairment, motor impairment, cognitive impairment, and swallowing disorders. Post-stroke depression (PSD) is of substantial concern due to its high morbidity, high disability rate, and poor response to treatment (Robinson and Jorge, 2016; Das J, 2018; Guo et al., 2022). It is the most frequent and burdensome neuropsychiatric complication following a stroke (Shi et al., 2017; Taylor-Rowan et al., 2019). Its clinical features include a depressed mood, loss of interest, and increased fatigue (Sun et al., 2024). Many factors contribute to the occurrence of PSD, including the history of depression, stroke severity, lesion location, and so on (Guo et al., 2022). Unfortunately, we lack a unified mechanism to explain PSD, the mechanisms of which now involve dysregulation of hypothalamic–pituitary–adrenal (HPA) axis, increased inflammatory factors, decreased levels of monoamines, glutamate-mediated excitotoxicity, and abnormal neurotrophic response (Guo et al., 2022).

In recent decades, the fields of microbiology and neuroscience have become increasingly intertwined. Bidirectional communication along the gut–brain axis is a fundamental mechanism for the development of gut–brain signaling pathways that link the microbiota with the host, influencing brain function and behavior (Rhee et al., 2009; Grenham et al., 2011; Cryan and Dinan, 2012; Mayer et al., 2015; McVey Neufeld et al., 2015; Dinan and Cryan, 2017). Recent research has shifted focus from gut–brain communication related to digestive function and satiety (Taché et al., 1980; Konturek et al., 2004; Berthoud, 2008) to its impact on higher-order cognition and psychological effects (Rhee et al., 2009; Carabotti et al., 2015; Sarkar et al., 2016; Agustí et al., 2018). The link between gut microbiota (GM) and central nervous system disorders, including Parkinson’s disease, cerebrovascular diseases, Alzheimer’s disease, autism spectrum disorder, and depression, has become a significant area of research. This has led to new insights into the pathogenesis and potential treatments for these central nervous system conditions (Cryan et al., 2020; Socała et al., 2021).

A large number of studies have shown that abnormal gut–brain networks not only exacerbate gut inflammatory disorders (Mayer et al., 2015; Bernstein, 2017; Breit et al., 2018) and altered acute and chronic stress responses (Dinan and Cryan, 2017; Foster et al., 2017; Marin et al., 2017; Gao et al., 2018; Maniscalco and Rinaman, 2018; Partrick et al., 2018; Sun et al., 2018; Provensi et al., 2019) but also affect behavioral states such as emotional well-being and motivated behavior, particularly anxiety-like behavior (Dinan and Cryan, 2017; Arentsen et al., 2018; Heintz-Buschart et al., 2018; Jaglin et al., 2018; Luk et al., 2018; Maniscalco and Rinaman, 2018; Fouhy et al., 2019). Evidence indicates that the composition and function of the GM are altered in patients with PSD (Jiang et al., 2021), suggesting a link between changes in intestinal flora and PSD. Therefore, exploring the brain–gut–microbiota axis may uncover fundamental mechanisms of PSD and contribute to the development of new antidepressant therapies. However, due to small sample sizes and inconsistent detection methods, most current studies on the GM in PSD exhibit considerable heterogeneity.

In this study, we conducted a meta-analysis of research comparing the GM between PSD patients and healthy controls or stroke groups. We searched eight public databases, extracting relevant data directly or indirectly from the articles. Finally, we evaluated the pooled differences in GM composition between PSD patients and the comparison groups.

## Materials and methods

### Search strategy

For the collection of eligible studies, we systematically and comprehensively searched in PubMed, Web of Science, Embase, Cochrane databases, Wangfang, VIP, CBM, and CNKI from the establishment of the database to April 17, 2024, with no language restriction. A detailed search strategy was developed using Medical Subject Headings (MeSH) and free terms. The MeSH words were as follows: “stroke”, “depression”, and “gastrointestinal microbiome”. This meta-analysis was conducted on the basis of the Preferred Reporting Items for Systematic Reviews and Meta-analyses (PRISMA) criteria (Page et al., 2021).Taking PubMed as an example, the search strategy is shown in **Supplementary Table S1**.

### Inclusion and exclusion criteria

Eligible studies were identified based on the following criteria (1): comparison of GM diversity and abundance between patients with PSD and healthy controls (HC) or stroke patients (2), GM samples were derived from stool (3), availability of adequate statistical data (e.g., mean, standard deviation, *p*-values, median, maximum, minimum, etc.) to estimate effect sizes, and (4) access to the full text. Case reports, systematic reviews, and animal research were excluded.

### Outcome measures

The primary outcomes consisted of GM diversity (including α diversity and β diversity) and differences of GM abundance between the patients with PSD and HC or stroke.

### Data extraction

For each eligible study, the following information was collected: the first authors’ name, date of publication, region, sample size, detection method, and richness and diversity indices from 16S rRNA sequencing data, including relative abundance of GM. Median, minimum, maximum, or 95% confidence intervals (CI) were estimated from histograms and box plots (Egger et al., 1997; Zuo et al., 2019; Huang et al., 2021; Kang et al., 2021; Li et al., 2021; Yao et al., 2023). Two authors (LQL and WXQ) independently extracted and assessed the data, with any disagreements resolved through consensus between the reviewers.

### Quality assessment

The quality and risk of bias were assessed by two authors (LQL and WXQ) independently. For quality assessment of cross-sectional studies, the Agency for Healthcare Research and Quality (AHRQ) criteria (Zeng et al., 2015) were used (Ma et al., 2023), which include 11 items scored as “yes”, “no”, or “unclear”. The total score ranges from 0 to 11 points, with scores of 0–3 indicating low quality, scores of 4–7 indicating medium quality, and scores greater than 8 indicating high quality (Zeng et al., 2015). Cohort studies were evaluated using the Newcastle–Ottawa Scale (NOS) (Ma et al., 2023), which has a maximum score of 9. Scores of 1 to 3 are classified as low quality, 4 to 6 as moderate quality, and 7 to 9 as high quality (Stang, 2010).

### Risk of bias assessment

For the risk of bias assessment, the Revised Risk of Bias Assessment Tool for Nonrandomized Studies (RoBANS 2) was used for each included study (Seo et al., 2023). This tool evaluates eight potential sources of bias: comparability of the target group, target group selection, confounders, measurement of intervention/exposure, blinding of assessors, outcome assessment, incomplete outcome data, and selective outcome reporting. Disagreements were resolved through consensus or by consulting with a third author.

### Effect size calculations

The Comprehensive Meta-Analysis Version 3 software (Biostat Inc., Englewood, NJ, USA) was applied to calculate the effect sizes with a random-effects, inverse-variance weighted model. Assuming that deviations from Gaussian distributions were minimal, we applied previously reported conversion equations (Hozo et al., 2005) to estimate means and standard deviation from median, maximum, and minimum. Hedges’ *g* effect sizes were calculated from the mean differences between PSD and HC or stroke groups, divided by the pooled standard deviation. Heterogeneity across studies was assessed using Q-statistic and *I* ^2^ statistic. Additionally, the presence of outliers could introduce bias and significantly affect the pooled effect sizes (Viechtbauer and Cheung, 2010; Noma et al., 2020). Outliers were defined by the following criteria (Medeiros et al., 2020): for which the upper boundary of the 95% CI was lower than the lower boundary of the overall effect CI (i.e., extremely small effect sizes) and (WHO, 2018a) for which the lower boundary of the 95% CI was higher than the upper boundary of the overall effect CI (i.e., extremely large effect sizes). To assess the robustness of our findings, we performed sensitivity analyses by sequentially excluding each study and recalculating the pooled effect size.

The potential publication bias of each GM abundance was quantitatively assessed by using Begg and Mazumdar rank correlation (Begg and Mazumdar, 1994) and Egger’s regression intercept tests (Egger et al., 1997). Moreover, the Duval and Tweedie’s trim and fill method was used to correct for non-normal distribution of effect sizes potentially due to the file drawer problem. The significant levels were set at *p <*0.05.

## Results

### Characteristics of eligible studies

**Figure 1:**
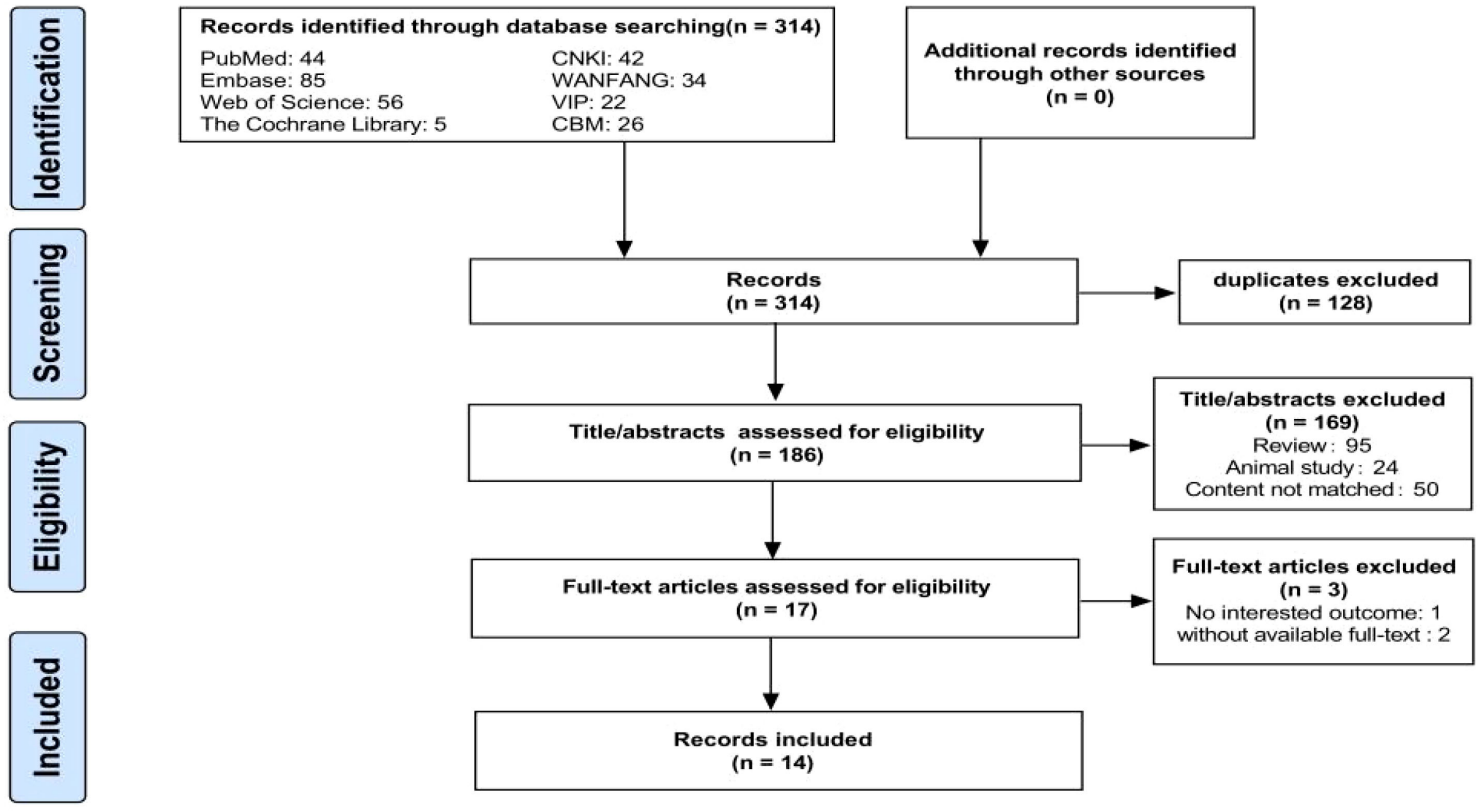
Flow chart of the search strategy and study selection process.

A flow chart of the literature search and selection process is shown in **Figure 1**. A total of 314 relevant articles were retrieved from the eight databases, with 128 being duplicates. Based on the titles and abstracts, 169 articles were excluded for not meeting the eligibility criteria. Subsequently, two studies were excluded due to unavailable full texts and one due to the lack of relevant outcomes, leaving 17 articles. Finally, 14 eligible articles were included in the meta-analysis (Fan et al., 2016; Sun, 2019; Zuo et al., 2019; Ling et al., 2020; Han, 2021; Huang et al., 2021; Kang et al., 2021; Li et al., 2021; Guo et al., 2022; Huang et al., 2022; Li et al., 2022; Qin et al., 2022; Wang, 2023; Yao et al., 2023). In totally, 1,556 individuals were included, comprising 702 patients with PSD, 512 with stroke, and 342 HC. Detailed information on the eligible articles is presented in **Table 1**. The 14 included studies were all conducted in China across nine provinces, with nine published in Chinese and five in English.

**Table 1:** Characteristics of each study included in the meta-analysis.

First author	Year	Province	Region/country	16S region	PSD/CTR	Female	Age	BMI	Hypertension	Diabetes mellitus	Dietary check	Seq. Tech.
Shanshan Yao	2023	Zhejiang	South/China	V3–V4	88/144^a^	41/43	65.43 ± 11.52/64.57 ± 11.81	NR	67/107	40/46	NR	Illumina MiSeq platform
Yingjia Wang	2023	Shanxi	North/China	V3–V4	28/26^a^	10/10	57.00 ± 5.89/55.88 ± 5.87	24.73 ± 2.71/ 23.82 ± 2.85	11/9	4/5	Yes	NovaSeq 6000
					28/22^b^	10/10	57.00 ± 5.89/54.50 ± 5.13	24.73 ± 2.71/ 21.57 ± 2.08	11/0	4/0	NR	
Xuebin Li	2022	Guangxi	South/China	V3–V4	26/34^a^	NR	NR	NR	NR	NR	NR	Illumina Miseq/HiSeq2500
Weicui Huang	2022	Liaoning	North/China	NR	87/126^a^	NR	NR	NR	NR	NR	NR	the Japanese Mitsuoka method
Xudong Guo	2022	Henan	North/China	V3	68/68^b^	32/28	52.5 ± 2.6/57.2 ± 1.8	NR	NR	NR	NR	Barcoded 454
Lihua Qin	2022	Henan	North/China	V3	46/46^b^	25/23	34.12 ± 1.22/35.22 ± 1.34	NR	NR	NR	NR	Barcoded 454
Yi Kang	2021	Chongqing	South/China	NR	67/96^a^	NR	NR	NR	NR	NR	NR	ATB-expression semi-automatic microbial detection system
Yinting Huang	2021	Guangdong	South/China	V3–V4	20/27^a^	NR	NR	NR	NR	NR	NR	MiSeq Benchtop Sequencer
Guangshun Han	2021	Guangxi	South/China	V3–V4	26/34^a^	10/14	58.38 ± 7.349/57.62 ± 7.394	21.19 ± 3.371/ 21.26 ± 3.184	NR	NR	NR	Illumina Miseq/HiSeq2500
					26/30^b^	10/13	58.38 ± 7.349/58.73 ± 6.357	21.19 ± 3.371/ 20.93 ± 2.912			NR	
Yanhong Li	2021	Henan	North/China	V3	76/76^b^	35/37	52.28 ± 2.35/51.84 ± 2.61	NR	NR	NR	NR	Barcoded 454
Yi Ling	2020	Zhejiang	South/China	V3–V4	41/25^a^	24/11	69.63 ± 9.35/68.92 ± 8.46	25.14 ± 3.62/ 26.62 ± 3.58	24/16	12/6	NR	MiSeq Benchtop Sequencer
Xinyue Sun	2019	Beijing	North/China	V3–V4	33/10^b^	18/6	72.20 ± 13.6/52.00 ± 4.70	NR	NR	NR	NR	Illumina MiseqPE
Xuecan Zuo	2019	Henan	North/China	V3	64/60^b^	28/26	52.3 ± 8.4/53.5 ± 8.6	NR	NR	NR	NR	Barcoded 454
Wentao Fan	2016	Shaanxi	North/China	V3	32/30^b^	NR	NR	NR	NR	NR	NR	Barcoded 454

PSD, post-stroke depression; CTR, control; NR, not reported; Seq. Tech., sequencing technology.

a PSD vs. stroke.

b PSD vs. HC.

The GM of 12 eligible studies was analyzed using high-throughput sequencing of the region of V3 or V3–V4 of the 16S rRNA gene. Specifically, Weicui Huang et al. detected and reported 12 components in GM using the Japanese Mitsuoka method (Huang et al., 2022). Yi Kang et al. only reported the presence of *Enterococcus faecalis*, *Escherichia coli*, and *Bifidobacterium* in feces using the ATB-expression semi-automatic microbial detection system (Kang et al., 2021). The methodological quality assessment revealed that seven studies (Zuo et al., 2019; Han, 2021; Guo et al., 2022; Li et al., 2022; Qin et al., 2022; Wang, 2023; Yao et al., 2023) had high quality, while the remaining seven (Fan et al., 2016; Sun, 2019; Ling et al., 2020; Huang et al., 2021; Kang et al., 2021; Li et al., 2021; Huang et al., 2022) were of fair quality. Details are provided in **Supplementary Tables S2-1** and **S2-2**.

### Primary outcomes: α diversity and β diversity

Among the indices of α diversity, Chao1 index, ACE indexes, Shannon index, and Simpson index were most frequently measured in the included studies. Heterogeneity testing was performed on these indices, with the results shown in **Table 2**. When comparing the α diversity between PSD spectrum and stroke groups, the heterogeneity test for the Chao1 index indicated *I*² = 59.287, while the Q test result showed that *P >*0.05. Consequently, a fixed-effects model was used for analysis. ACE index, Shannon index, and Simpson index exhibited low heterogeneity and were also analyzed using the fixed-effects model. For comparisons of α diversity between PSD spectrum and HC, the evenness index showed *I* ^2^ = 54.933, with the Q test result indicating *P >*0.05; thus, a fixed-effects model was applied. However, Chao1 index, ACE index, Shannon index, and Simpson index all demonstrated high heterogeneity. Therefore, a random-effects model was used for these analyses. Simultaneously, moderation effect analysis was conducted to investigate the sources of heterogeneity among the research results. After excluding a study with a sample size of less than 50, Chao1 index, ACE indexes, and Shannon index between PSD spectrum and HC changes were substantial in the effect size, suggesting a large and statistically significant effect. Nevertheless, the effect sizes of other indexes did not show obvious changes (**Supplementary Table S5**).

**Table 2:** Heterogeneity analysis and main effect analysis statistical results of α diversity.

α diversity	*k*	Heterogeneity							Hedges’ g
					95% CI		Test of null (two-tail)		
		Q	*P*-value	*I* ^2^	Lower limit	Upper limit	Z-value	*P*-value	
Random effects model									
PSD vs. HC									
Chao1	5	93.039	0.000	95.701	-0.100	2.184	1.788	0.074	1.042
ACE	6	59.617	0.000	91.613	-0.189	1.191	1.423	0.155	0.501
Shannon	6	155.880	0.000	96.792	-0.571	1.773	1.005	0.315	0.601
Simpson	5	32.880	0.000	87.835	0.057	1.271	2.143	0.032	0.664
Fixed effects model									
Evenness	3	4.438	0.109	54.933	-0.421	0.015	-1.824	0.068	-0.203
PSD vs. stroke									
Chao1	3	4.912	0.086	59.287	-0.170	0.268	0.438	0.661	0.049
ACE	3	2.215	0.330	9.706	-0.572	0.046	-1.669	0.095	-0.263
Shannon	5	1.564	0.815	0.000	-0.212	0.159	-0.284	0.776	-0.027
Simpson	3	1.400	0.497	0.000	-0.672	-0.059	-2.334	0.020	-0.365

**Figure 2:**
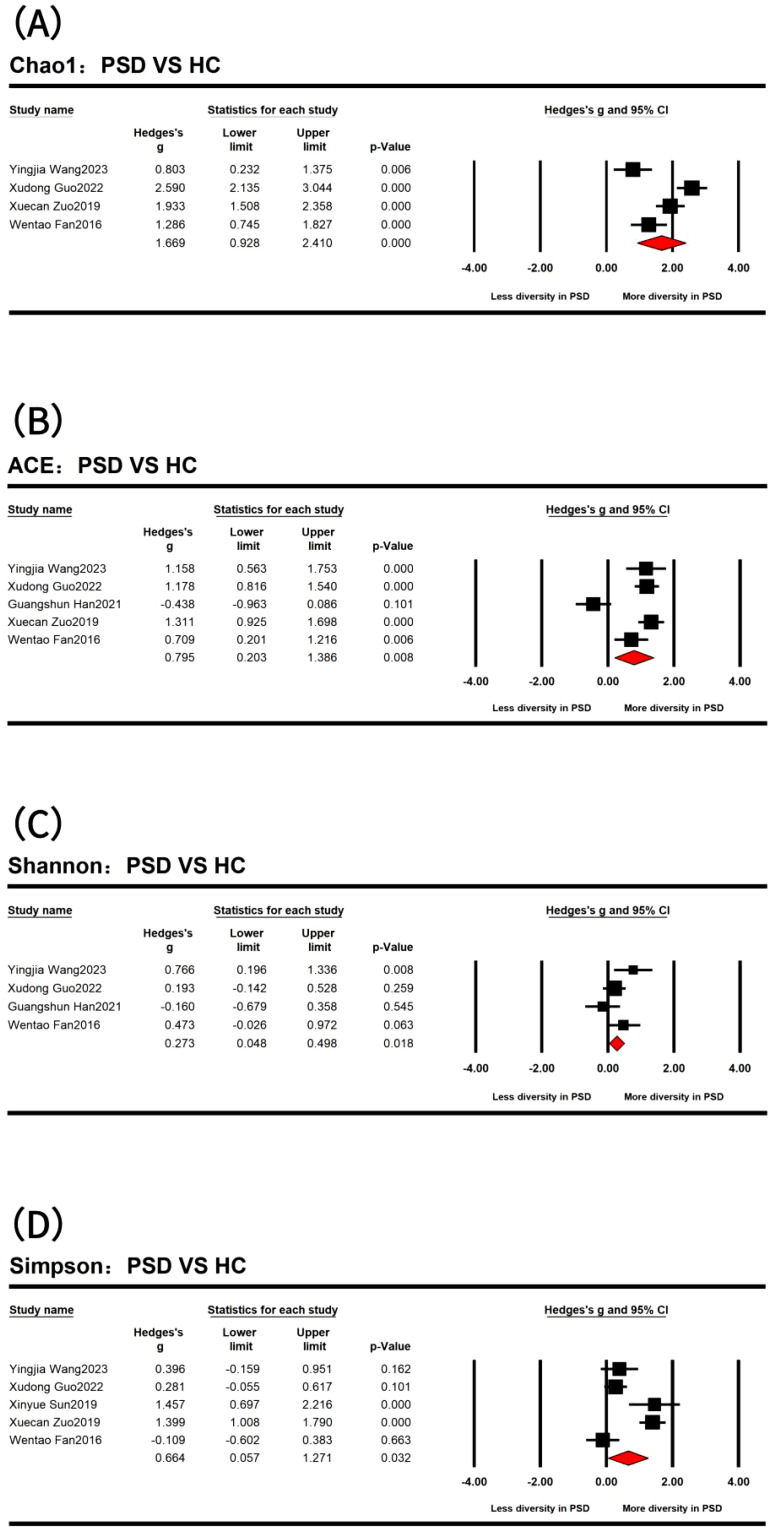
Forest plots of Chao1 index **(A)**, ACE indexes **(B)**, Shannon index **(C)**, and Simpson index **(D)** in the comparisons between post-stroke depression (PSD) and healthy controls (HC) spectrum.

In the comparison of α diversity between PSD spectrum and stroke, only the Simpson index showed a statistical difference (**Supplementary Figure S1**). Specifically, the stroke spectrum demonstrated significantly increased α diversity as indicated by the Simpson index (Hedges’ *g* = -0.365; 95% CI = -0.672 to -0.059; *p* = 0.020; *n* = 3). However, when comparing the PSD spectrum and HC and after removing several outlier results (**Figure 2**), the PSD spectrum demonstrated significantly increased α diversity as indicated by the Chao1 index (Hedges’ *g* = 1.669; 95% CI = 0.928 to 2.410; *p* < 0.001; *n* = 4), ACE indexes (Hedges’ *g* = 0.795; 95% CI = 0.203 to 1.386; *p* = 0.008; *n* = 5), Shannon index (Hedges’ *g* = 0.273; 95% CI = 0.048 to 0.498; *p* = 0.018; *n* = 4), and Simpson index (Hedges’ *g* = 0.664; 95% CI = 0.057 to 1.271; *p* = 0.032; *n* = 5). Additionally, the results of the three studies that compared the evenness index between the PSD spectrum and HC showed no significant differences between the two groups.

Only four (Ling et al., 2020; Huang et al., 2021; Li et al., 2022; Wang, 2023) of the included articles assessed β diversity (**Table 3**). The principal coordinate analyses were based on weighted UniFrac distance, Bray–Curtis dissimilarity, and partial least squares discriminant analysis (PLS-DA). Regarding differences between PSD and stroke patients, three studies found significant differences (Huang et al., 2021; Li et al., 2022; Wang, 2023), while one study (Ling et al., 2020) did not. Additionally, two (Li et al., 2022; Wang, 2023) studies reported significant differences between the PSD spectrum and HC. In summary, the findings across the included studies were inconsistent.

**Table 3:** Summary of β diversity assessments in the included studies.

Study	β diversity	Find	Statistic value
Yingjia Wang	PCoA based on Bray–Curtis dissimilarity	A significant difference in gut microbial composition between PSD and stroke	*P* = 0.001
	PCoA based on Bray–Curtis dissimilarity	A significant difference in gut microbial composition between PSD and HC	*P* = 0.001
Xuebin Li	PCoA of weighted Unifrac distances	A significant difference in gut microbial composition between PSD and stroke	NR
	PCoA of weighted Unifrac distances	A significant difference in gut microbial composition between PSD and HC	NR
Yinting Huang Yi Ling	PLS-DA	A clear difference in gut microbial composition between PSD and stroke	NR
	PCoA based on Bray–Curtis dissimilarity	No significant difference in gut microbial composition among PSD and stroke	*P* = 0.384

PSD, post-stroke depression; HC, health control; NR, not reported; PCoA, principal coordinate analysis; PLS-DA, partial least squares discriminant analysis.

### Primary outcomes: differences in microbial composition at the phylum level

**Figure 3:**
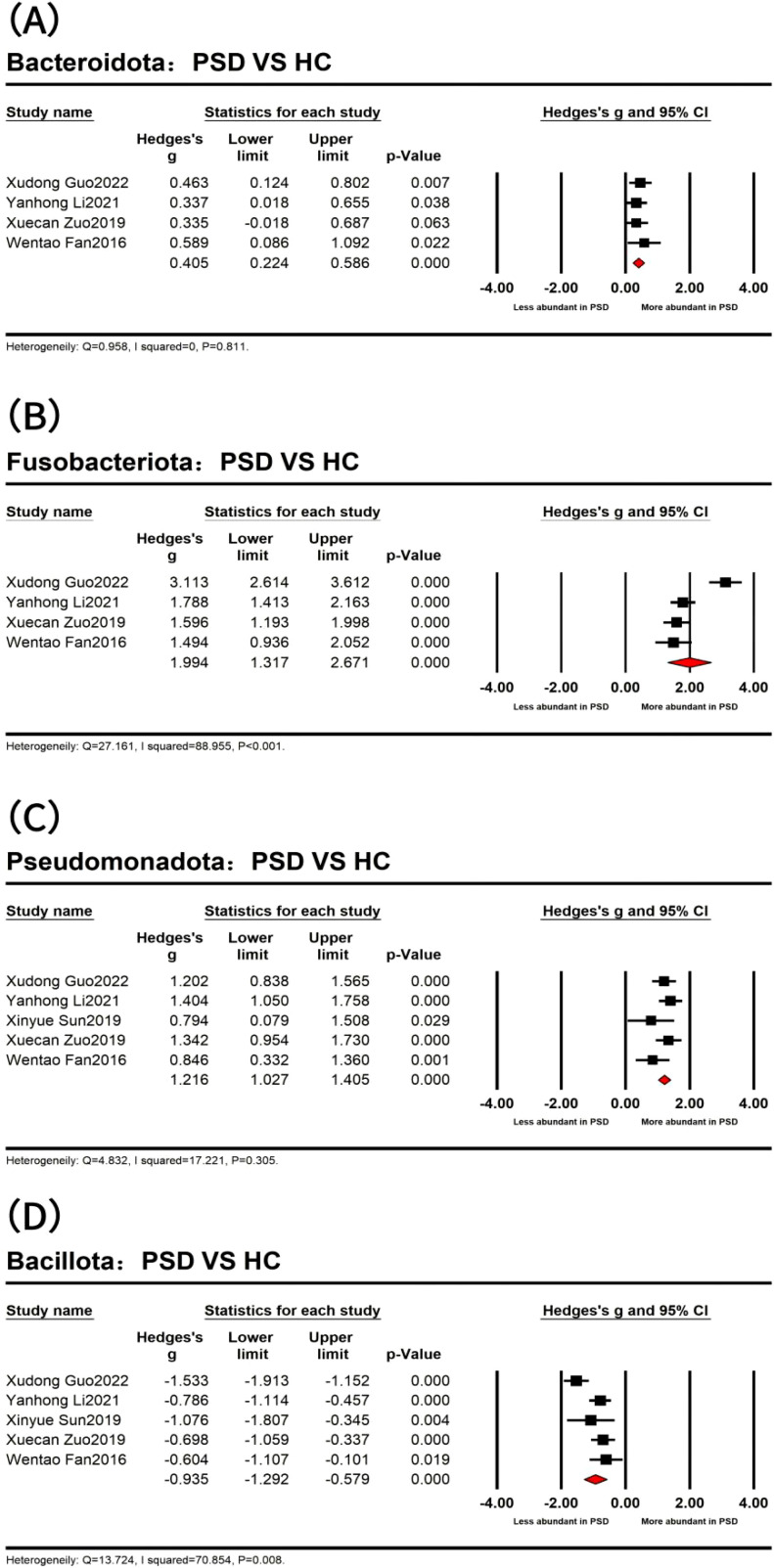
Forest plots of alterations of gut microbiota at the phylum level between post-stroke depression (PSD) and healthy controls (HC) spectrum, including *Bacteroidota* **(A)**, *Fusobacteriota* **(B)**, *Pseudomonadota* **(C)**, and *Bacillota* **(D)**.

At the phylum level (**Figure 3**), the results indicated that four dominant bacterial phyla were identified across 273 PSD spectrum and 244 HC in five studies (Fan et al., 2016; Sun, 2019; Zuo et al., 2019; Li et al., 2021; Guo et al., 2022). Specifically, the analyses of 240 PSD spectrum and 234 HC from four studies (Fan et al., 2016; Zuo et al., 2019; Li et al., 2021; Guo et al., 2022) revealed a significantly higher abundance of *Bacteroidota* (*Bacteroidetes*) (Hedges’ *g* = 0.405; 95% CI = 0.224 to 0.586; *p* < 0.001; *n* = 4) and *Fusobacteriota* (*Fusobacteria*) (Hedges’ *g* = 1.994; 95% CI = 1.317 to 2.671; *p* < 0.001; *n* = 4) in the PSD spectrum compared to HC. The remaining two dominant bacterial phyla were *Pseudomonadota* (*Proteobacteria*) and *Bacillota* (*Firmicutes*), with significant statistical differences observed in the five studies. Specifically, *Pseudomonadota* was more abundant in the PSD spectrum (Hedges’ *g* = 1.216; 95% CI = 1.027 to 1.405; *p* < 0.001; *n* = 5), while *Bacillota* was significantly less abundant in the PSD spectrum (Hedges’ *g* = -0.935; 95% CI = -1.292 to -0.579; *p* < 0.001; *n* = 5). However, no meaningful phylum-level differences were observed between PSD spectrum and stroke in the included studies. The heterogeneity test results showed that the *I* ^2^ of *Bacteroidota* and *Pseudomonadota* was <75%, besides the Q test with *P >*0.05. The fixed effect model was used, while the random effects model was used for *Bacillota* and *Fusobacteriota*. Details are provided in **Table 4**.

**Table 4:** Heterogeneity analysis and main effect analysis statistical results of gut microbiota at the phylum level.

Phylum level	*k*	Heterogeneity							Hedges’ g
					95% CI		Test of null (two-tail)		
		Q	*P*-value	*I* ^2^	Lower limit	Upper limit	*Z*-value	*P*-value	
Random effects model									
*Bacillota*	5	13.724	0.008	70.854	-1.292	-0.579	-5.141	0.000	-0.935
*Fusobacteriota*	4	27.161	0.000	88.955	1.317	2.671	5.772	0.000	1.994
Fixed effects model									
*Bacteroidota*	4	0.958	0.811	0.000	0.224	0.586	4.386	0.000	0.405
*Pseudomonadota*	5	4.832	0.305	17.221	1.027	1.405	12.605	0.000	1.216

### Primary outcomes: differences in microbial composition at the family level

At the family level (**Table 5**), a total of 11 dominant bacterial families were identified in 240 PSD spectrum and 234 HC across four studies (Fan et al., 2016; Zuo et al., 2019; Li et al., 2021; Guo et al., 2022). The combined results of *Brassicaceae* were derived from three studies involving 208 PSD spectrum and 204 HC. The heterogeneity test results indicated that *Prevotellaceae*, *Ruminococcaceae*, and *Lachnospiraceae* had *I* ^2^ values of <75%, besides Q test *P*-values >0.05, so these were analyzed using a fixed effects model. In contrast, the remaining eight dominant bacterial families exhibited high heterogeneity and were analyzed using random effects model. Specifically, increased abundances of *Rikenellaceae*, *Acidaminococcaceae*, *Fusobacteriaceae*, *Brassicaceae*, *Enterobacteriaceae*, *Porphyromonadaceae*, and *Erysipelotrichaceae* were observed in the PSD spectrum compared to HC. Conversely, *Prevotellaceae*, *Ruminococcaceae*, and

**Table 5:** Heterogeneity analysis and main effect analysis statistical results of gut microbiota at the family level.

Family level	*k*	Heterogeneity							Hedges’ g
					95% CI		Test of null (two-tail)		
		Q	*P*-value	*I* ^2^	Lower limit	Upper limit	*Z*-value	*P*-value	
Random effects model									
*Rikenellaceae*	4	165.720	0.000	98.190	4.653	10.705	4.973	0.000	7.679
*Acidaminococcaceae*	4	228.190	0.000	98.685	6.168	12.557	5.744	0.000	9.363
*Fusobacteriaceae*	4	80.269	0.000	96.263	1.988	4.953	4.589	0.000	3.470
*Brassicaceae*	3	74.509	0.000	97.316	2.156	6.449	3.929	0.000	4.303
*Enterobacteriaceae*	4	253.134	0.000	98.815	1.843	8.517	3.043	0.002	5.180
*Porphyromonadaceae*	4	51.825	0.000	94.211	2.782	5.416	6.100	0.000	4.099
*Erysipelotrichaceae*	4	222.155	0.000	98.650	-6.677	-1.806	-3.413	0.001	-4.241
*Bacteroidaceae*	4	169.087	0.000	98.226	-0.890	2.159	0.816	0.415	0.635
Fixed effects model									
*Prevotellaceae*	4	0.203	0.977	0.000	-0.837	-0.470	-6.973	0.000	-0.654
*Ruminococcaceae*	4	6.098	0.107	50.806	-2.147	-1.712	-17.373	0.000	-1.930
*Lachnospiraceae*	4	0.143	0.986	0.000	-0.622	-0.260	-4.773	0.000	-0.441

*Lachnospiraceae* were significantly less abundant in the PSD spectrum compared to HC. After excluding one outlier for *Bacteroidaceae*, the heterogeneity test revealed *I*² = 0 and Q test *p >*0.05, allowing for re-analysis using a fixed effects model, which showed a reduced abundance of *Bacteroidaceae* (Hedges’ *g* = -0.240; 95% CI = -0.458 to -0.023; *p* = 0.031; *n* = 3) in the PSD spectrum compared to HC.

### Primary outcomes: differences in microbial composition at the genus level

At the genus level (**Table 6**), a total of 21 dominant bacterial genera were identified. Of these, 19 were compared between PSD spectrum and HC, while two were compared between PSD spectrum and stroke. Firstly, a heterogeneity test was performed. For the 19 bacterial genera analyzed between PSD spectrum and HC, the results showed that the *I* ^2^ values for *Parasutterella*, *Parabasalids*, *Clostridium*, and *Alisma* were <75%, while the Q test indicated *P >*0.05. Therefore, a fixed effects model was used for these analyses. The remaining dominant bacterial genera exhibited high heterogeneity and were analyzed using a random effects model for comparisons between PSD spectrum and stroke. The heterogeneity test results showed that *Escherichia coli* had an *I* ^2^ value >75% and a Q test *p*-value <0.001, so a random effects model was employed. Conversely, for *Lachnospira*, the *I* ^2^ value was 0 and *p* > 0.05, so a fixed effects model was used.

**Table 6:** Heterogeneity analysis and main effect analysis statistical results of gut microbiota at the genus level.

Genus level	*k*	Heterogeneity							Hedges’ g
					95% CI		Test of null (two-tail)		
		Q	*P*-value	*I* ^2^	Lower limit	Upper limit	*Z*-value	*P*-value	
Random effects model									
*Bacillus anthracis*	3	15.672	0.000	87.238	2.603	4.312	7.929	0.000	3.457
*Escherichia*/*Shigella*	4	48.050	0.000	93.756	1.682	3.732	5.176	0.000	2.707
*Clostridium* XIX	2	103.673	0.000	99.035	-1.743	6.242	1.104	0.270	2.249
*Roseburia*	5	244.041	0.000	98.361	-0.163	4.059	1.808	0.071	1.948
*Blautia*	4	195.405	0.000	98.465	1.228	6.052	2.958	0.003	3.640
*Lachnospira*	3	154.901	0.000	98.709	-8.731	0.785	-1.637	0.102	-3.973
*Bacteroides*	2	114.610	0.000	99.127	-4.126	3.821	-0.075	0.940	-0.152
*Alistipes*	2	7.080	0.008	85.876	3.380	6.454	6.271	0.000	4.917
*Megamonas*	4	39.262	0.000	92.359	0.759	2.260	3.944	0.000	1.510
*Prevotella*	4	26.377	0.000	88.626	-2.330	-1.053	-5.192	0.000	-1.692
*Ruminococcus*	4	68.620	0.000	95.628	-5.271	-2.382	-5.192	0.000	-3.827
*F. prausnitzii*	4	248.428	0.000	98.792	-0.201	4.651	1.798	0.072	2.225
*Curtobacterium*	4	115.509	0.000	97.403	-4.984	-1.684	-3.961	0.000	-3.334
*Lacuna*	2	9.838	0.002	89.835	0.720	2.419	3.622	0.000	1.570
*Paraptera*	2	33.728	0.000	97.035	0.315	3.756	2.318	0.020	2.035
Fixed effects model									
*Parasutterella*	2	3.419	14.547	0.000	0.772	0.380	0.000	2.607	3.013
*Parabasalids*	2	1.776	11.099	0.000	0.881	0.348	0.000	1.243	1.510
*Clostridium*	2	2.454	14.279	0.000	1.352	0.245	26.049	1.861	2.157
*Alisma*	2	5.468	20.406	0.000	3.191	0.074	68.661	4.510	4.989

*Lachnospira* (Hedges’ *g* = -1.650; 95% CI = -1.964 to -1.336; *p* < 0.001; n = 2). However, the combined effect of *Escherichia coli* in the PSD spectrum versus stroke was not statistically significant.

In detail, the PSD spectrum exhibited significantly higher levels of *Bacillus anthracis*, *Escherichia*/*Shigella*, *Blautia*, *Alistipes*, *Megamonas*, *Parasutterella*, *Parabasalids*, *Lacuna*, *Clostridium*, *Paraptera*, and *Alisma* compared to HC. After excluding one outlier, significantly higher abundances were also found for *Roseburia* (Hedges’ *g* = 3.211; 95% CI = 1.603 to 4.818; *p* < 0.001; *n* = 4), *Lachnospira* (Hedges’ *g* = 3.364; 95% CI = 0.222 to 6.506; *p* = 0.036; *n* = 2), and *Faecalibacterium prausnitzii* (*F. prausnitzii*) (Hedges’ *g* = 3.477; 95% CI = 2.128 to 4.826; *p* < 0.001; *n* = 3) in the PSD spectrum compared to HC. Conversely, the levels of *Prevotella*, *Ruminococcus*, and *Curtobacterium* were significantly lower in the PSD spectrum than in HC. Additionally, no significant differences were observed for *Clostridium* XIX and *Bacteroides* between the two groups. In comparisons between the PSD spectrum and stroke spectrum, the PSD spectrum had significantly lower levels of *Lachnospira* (Hedges’ g = -1.650; 95% CI = -1.964 to -1.336; p < 0.001; n = 2). However, the combined effect of *Escherichia coli* in the PSD spectrum versus stroke was not statistically significant.

### Risk of bias

The quality of the included studies is summarized in **Supplementary Table S3**. Each study was classified as low risk for seven criteria. In addition, regarding confounding bias, only Yingjia Wang^2023^ controlled important confounding factors, such as BMI, diabetes, and diet, rated “low risk”. The remaining 13 studies all had unmeasured confounding factors, all rated “moderate risk”.

### Publication bias

The Begg and Mazumdar rank correlations tests, along with Egger’s regression intercept tests, indicated that most of these meta-analysis results were not significantly biased by publication errors, with the exception of F-Porphyromonadaceae. The adjusted Hedges’ *g* was calculated for eight GM strains: F-Acidaminococcaceae, F-Fusobacteriaceae, F-Brassicaceae, F-Enterobacteriaceae, G-Blautia, G-Megamonas, G-Prevotella, and G-Faecalibacterium (**Supplementary Table S4**).

## Discussion

This meta-analysis compared the GM abundance between patients with PSD spectrum and HC or stroke, providing five key insights into GM alterations associated with the PSD spectrum. First, α diversity was significantly higher in the PSD spectrum compared to HC but showed no significant difference when compared to the stroke spectrum. Second, the relative abundance of *Pseudomonadota*, *Bacteroidota*, and *Fusobacteriota* was higher in the PSD spectrum compared to HC, whereas *Bacillota* were less abundant. Third, at the family level, six bacterial families were more abundant and five were less abundant in the PSD spectrum compared to HC. Fourth, at the genus level, 14 genera were more abundant and five were less abundant in the PSD spectrum compared to HC. Finally, *Lachnospira* was found to be less abundant in the PSD spectrum compared to the stroke spectrum.

To our knowledge, previous meta-analyses have only evaluated α diversity and β diversity between the PSD spectrum and HC. Our study is the first to incorporate comparisons between the PSD spectrum and stroke spectrum. Our findings revealed that the Simpson index diversity was significantly higher in the stroke spectrum compared to the PSD spectrum, while other results showed no significant statistical differences. However, when comparing the PSD spectrum to the HC spectrum, we observed a significant increase in α diversity within the PSD spectrum. This finding contrasts with the results reported by Fang Luo et al (Luo and Fang, 2022). Regarding β diversity, the studies included in our meta-analysis yielded inconsistent results, warranting further investigation. Although increased gut microbial diversity is generally viewed as an indicator of health, it can also occur in the presence of potentially pathogenic and pro-inflammatory bacteria (Frost et al., 2019; Simpson et al., 2021). Therefore, the observed increase in gut microbial diversity within the PSD spectrum may reflect a similar underlying mechanism. The results of the sensitivity analyses showed that substantial changes in the effect size following the exclusion of a small-sample study highlight the sensitivity of our results to the inclusion of such studies. This sensitivity suggests that the original pooled effect size may underestimate the true effect. Small-sample studies are more susceptible to bias due to limited statistical power and potential methodological limitations, which could lead to either an overestimation or underestimation of the true effect. To enhance the robustness of future meta-analyses, we recommend prioritizing studies with larger sample sizes and standardized methodologies. Furthermore, sensitivity analyses should be routinely conducted to evaluate the influence of small-sample studies on pooled estimates and to ensure the reliability of the findings.

The phylum *Bacillota* is a significant group of bacteria in the human body, predominantly composed of gram-positive species. Most members of the *Bacillota* phylum are beneficial bacteria. For example, *Lactobacillus* species produce acetate and lactate. Moreover, antibacterial substances help prevent pathogen-related health disturbances. Butyrate, a crucial short-chain fatty acid (SCFA), has several important physiological roles, including trans-epithelial transport, reduction of mucosal inflammation, alleviation of oxidative stress, enforcement of the epithelial barrier, and further protection against colorectal cancer (CRC) (Hamer et al., 2008). Butyrate-synthesizing bacteria are predominantly found within the *Bacillota* phylum (Singh et al., 2023). Additionally, *Bacillota* is associated with anti-inflammatory effects, modulation of metabolic functions, and also SCFA production (Kumar et al., 2014; Welcome, 2019). There is also a positive correlation between *Bacillota* abundance and executive function performance, indicating that this phylum constitutes a beneficial component of the gut microbiota (Bhat and Kapila, 2017; Sheng et al., 2021). Our study results indicate that the abundance of *Bacillota* is lower in the PSD spectrum compared to the HC spectrum. Within the *Bacillota* phylum, *Lachnospiraceae*, *Ruminococcaceae*, *Eubacteriaceae*, and *Clostridiaceae* are the four important butyrate-synthesizing families (Singh et al., 2023). The results of our study found that members of *Bacillota*, including *Lachnospiraceae*, *Ruminococcaceae*, *Erysipelotrichaceae*, *Ruminococcus*, and *F. prausnitzii* also showed a decrease in abundance within the PSD spectrum compared with HC.

*Pseudomonadota* is a major phylum of gram-negative bacteria (Zhao and Lukiw, 2018) that includes a diverse range of metabolic species. Numerous studies have shown that an increase in intestinal *Pseudomonadota* is indicative of microbial dysbiosis or an unstable intestinal microbial community structure (Shin et al., 2015). Our study also found a significant increase in *Pseudomonadota* abundance within the PSD spectrum. Specifically, bacterial groups within *Pseudomonadota*, such as *Enterobacteriaceae*, *Lachnospira*, and *Escherichia/Shigella*, were more abundant in the PSD spectrum. Notably, neurotoxins derived from *Escherichia coli*, a member of the *Pseudomonadota* phylum, are known to enhance the release of proinflammatory cytokines (Cattaneo et al., 2017). Furthermore, increased *Pseudomonadota* abundance has been associated with worsening memory dysfunction (Hossain et al., 2019; Jeong et al., 2019). In summary, our finding of abnormally high *Pseudomonadota* levels in the PSD spectrum is consistent with previous literature.

*Bacillota* (formerly known as *Firmicutes*) and *Bacteroidota* are the two dominant phyla in the gut, comprising approximately 90% of the total gut microbiota (Human Microbiome Project Consortium. Structure, function and diversity of the healthy human microbiome, 2012). The *Firmicutes*/*Bacteroidetes* (F/B) ratio is an important indicator of gut microbiota health (Li and Ma, 2020); even deviations in this ratio are associated with various pathological conditions, including age-related disorders. An increase or decrease in the F/B ratio is often a sign of intestinal dysbiosis (Liang et al., 2018; An et al., 2023). An increased F/B ratio is commonly linked to obesity or metabolic disorders, potentially due to enhanced caloric extraction from food, fat deposition, lipogenesis, or impaired insulin sensitivity (Gibiino et al., 2018; Grigor’eva, 2020; Stojanov et al., 2020; Vaiserman et al., 2020). Conversely, a decreased F/B ratio is associated with conditions such as inflammatory bowel disease and depression as well as Alzheimer’s disease. This may result from an immune-inflammatory response triggered by reduced short-chain fatty acids (especially butyrate) production as well as the accumulation of protein metabolite like histamine and lipopolysaccharide (Gibiino et al., 2018; Schoeler and Caesar, 2019; Stojanov et al., 2020; Vaiserman et al., 2020).

Our study found that the phylum *Bacteroidota* was significantly more abundant in the PSD spectrum compared to HC, while the phylum *Bacillota* was reduced, resulting in a decreased F/B ratio indicative of intestinal dysbiosis. This finding aligns with the research by Lanxiang Liu et al., where intestinal dysbiosis was shown as a reduced F/B ratio. It is consistent with the research results of Lanxiang Liu et al (Liu et al., 2023). on major depressive disorder (MDD), which also reported a reduced F/B ratio in depressed patients. In addition, our study observed that at the family and genus levels, the abundance of *Prevotellaceae*, *Bacteroidaceae*, *Bacteroides*, and *Prevotella* was lower in the PSD spectrum compared to HC, which contrasts with the overall trends seen at the phylum level. This discrepancy be related to the intestinal barrier damage together with increased permeability, leading to bacterial translocation. Notably, previous studies have identified Alloprevotella as more dominant in healthy controls than in depressed patients (Zheng et al., 2021), consistent with our findings. Furthermore, research by Agirman et al (Agirman et al., 2021). indicated that increased intestinal permeability in depressed patients can lead to elevated plasma lipopolysaccharide (LPS) levels, which compromise the integrity of the blood–brain barrier (BBB). This breach allows inflammatory factors along with neurotoxins to enter the brain, activating immune cells and triggering inflammatory responses that contribute to the development of depression (Matsuno et al., 2022).

The phylum *Fusobacteriota* predominantly inhabits the oral cavity and colon, with certain species acting as opportunistic pathogens that can cause bacteremia together with various rapidly progressive infections. The virulence of *Fusobacteriota* is likely due to the production of lipopolysaccharide (LPS) and endotoxins as well as hemolysin (Brennan and Garrett, 2019). LPS can activate the TLR4-mediated NF-κB signaling pathway, leading to the production of pro-inflammatory cytokines such as interleukin-6 (IL-6), interleukin-8 (IL-8), and tumor necrosis factor-α (TNF-α) (Rubinstein et al., 2013; Abed et al., 2016; Bullman et al., 2017; Yang et al., 2017). A cross-sectional study investigating oral microbiota and ischemic stroke risk in elderly Chinese women revealed that specific imbalances in oral microbial composition (e.g., reduced *Streptococcus* while increased *Porphyromonas*) may elevate stroke risk through pro-inflammatory mechanisms (Wang et al., 2023). Through multi-omics analyses of early-onset cryptogenic stroke, Manzoor et al. demonstrated the elevated oral abundance of *Fusobacterium nucleatum* (*Fusobacteria* phylum), which established symbiotic interactions with pro-inflammatory *Candida* species, potentially promoting stroke pathogenesis via enhanced inflammatory responses along with thrombotic pathways (Manzoor et al., 2024).

Our study found that the abundance of the phylum *Fusobacteriota* was significantly higher in the PSD spectrum, with a corresponding increase in the *Fusobacteriaceae* family compared to HC. This heightened presence of *Fusobacteriota* may contribute to the development of post-stroke disease and could also play a role in the onset of post-stroke depression.

In addition, when comparing the gut microbiota between the PSD spectrum and stroke spectrum, no significant differences were observed in most bacterial groups, with *Lachnospira* being the only genus significantly less abundant in the PSD spectrum compared to the stroke spectrum (Huang et al., 2022; Li et al., 2022; Wang, 2023; Yao et al., 2023). This finding supports the earlier conclusion that *Lachnospiraceae*, a key butyrate-synthesizing family within the phylum *Bacillota*, may play a crucial role in the development of post-stroke depression due to its reduced presence in the PSD spectrum. A study investigating the gut microbiota in patients with mild cognitive impairment (MCI) and Alzheimer’s disease (AD) found that, compared to healthy controls, both AD and MCI patients exhibited a trend toward decreased *Bacteroides*, *Lachnospira*, and *Ruminiclostridium_9* instead of an increase in *Prevotella* (Guo et al., 2021). Among these, *Lachnospira* was the only genus that showed a statistically significant reduction in MCI patients compared to healthy controls, highlighting its potential importance (Wang et al., 2023). Moreover, research has indicated that *Lachnospira*, along with *Roseburia* and *Faecalibacterium*, is negatively correlated with the severity of depressive symptoms. This underscores the significant role of *Lachnospira* in depression, suggesting that it could become a focal point for future research on the gut microbiota in stroke and PSD patients (Ye et al., 2021). However, given the current scarcity of studies comparing the gut microbiota between the PSD spectrum and stroke, further research is needed to draw more definitive conclusions so as to develop a more precise treatment for PSD.

It is worth noting that attributing to the variability in reporting standards across studies, as well as the lack of consistent measurement methods for certain confounders in the existing literature, the incomplete control of confounding factors exists universally. The potential impact of unmeasured confounders, such as diet, medication, and other health conditions, cannot be ruled out. These factors may have led to an overestimation/underestimation of the observed effect size. Given the comparability of baseline data, we still conducted a pooled analysis of the research results. Thus, the results of this meta-analysis should be interpreted with caution. Meanwhile, future studies are encouraged to adopt standardized reporting of confounders and employ more rigorous methods to control for these variables. To enhance the quality of future meta-analyses, we recommend that primary studies provide a more detailed reporting of confounding factors as well as their adjustment methods. Furthermore, the development of standardized guidelines for measuring and reporting confounders would significantly improve the comparability and reliability of pooled estimates.

### Limitations

Despite these intriguing findings, our study has several limitations. First, we manually extracted data from the bar and box plots in several studies, which may introduce some biases. However, this process was conducted independently by two authors, reducing the likelihood that this would substantially affect the direction of statistical significance in our comparisons. Second, variations in sequencing methods could also contribute to potential biases in the results. For instance, differences in GM diversity between groups might be more pronounced when analyzing the V3–V4 region compared to the V3 region alone. However, the limited number of studies (five for the V3 region and seven for the V3–V4 region) restricted our ability to perform additional analyses. Third, all of the data were derived from studies conducted in China, involving a limited number of cities (nine provinces), which raises concerns about the generalizability of these findings to other populations.

## Conclusion

In summary, our study found that the α diversity in PSD patients was higher than in HC, with notable differences in the distribution of GM at the phylum, family, and genus levels. Additionally, the abundance of *Lachnospira* was lower in PSD patients compared to that in the stroke group. However, due to the limited number of studies, small sample size, and geographical constraints, besides the inconsistent sequencing technologies, these findings should be interpreted with caution and may not be generalizable to a broader population. Future studies with higher quality as well as a wider scope are necessary to validate these results.

## Data Availability

The original contributions presented in the study are included in the article/**Supplementary Material**. Further inquiries can be directed to the corresponding author.
