# Supplementary material for: Gut microbiota of patients with post-stroke depression in Chinese population: a systematic review and meta-analysis
Source: Front Cell Infect Microbiol. 2025 May 1;15:1444793. doi: 10.3389/fcimb.2025.1444793 (PMC12078233; doi:10.3389/fcimb.2025.1444793)
Supplement: Supplementary file 1 [file DataSheet1.pdf]

Supplementary Table Abstract

Gut microbiota of patients with post-stroke depression in Chinese population:a systematic review and meta-analysis

content

Supplementary table 1. Search strategies of PubMed databases. 1

Supplementary table 2-1. Quality of Cohort studies included in the meta-analysis (NOS) 3

Supplementary table 2-2. Quality of Cross-sectional studies included in the meta-analysis (AHRQ) 3

Supplementary table 3. Bias of the 14 studies included in this meta-analysis based on RoBANS. 4

Supplementary table 4. Publication bias assessments. 6

Supplementary table 5. Heterogeneity analysis and main effect analysis statistical results of α diversity after remove a excluding a study. 8

Supplementary Figure 1. Forest plots of Chao1 index (A); ACE indexes (B); Shannon index (C) and Simpson index (D) in the comparisons between post-stroke depression (PSD) and healthy controls (HC) spectrum. 9

Supplementary table 1. Search strategies of PubMed databases.

Number	Search Strategies
#1	((((((((((((((((((((((((((((stroke) OR (strokes)) OR (Cerebrovascular Accident)) OR (Cerebrovascular Accidents)) OR (CVA (Cerebrovascular Accident))) OR (CVAs (Cerebrovascular Accident))) OR (Cerebrovascular Apoplexy)) OR (Apoplexy, Cerebrovascular)) OR (Vascular Accident, Brain)) OR (Brain Vascular Accident)) OR (Brain Vascular Accidents)) OR (Vascular Accidents, Brain)) OR (Cerebrovascular Stroke)) OR (Cerebrovascular Strokes)) OR (Stroke, Cerebrovascular)) OR (Strokes, Cerebrovascular)) OR (Apoplexy)) OR (Cerebral Stroke)) OR (Cerebral Strokes)) OR (Stroke, Cerebral)) OR (Strokes, Cerebral)) OR (Stroke, Acute)) OR (Acute Stroke)) OR (Acute Strokes)) OR (Strokes, Acute)) OR (Cerebrovascular Accident, Acute)) OR (Acute Cerebrovascular Accident)) OR (Acute Cerebrovascular Accidents)) OR (Cerebrovascular Accidents, Acute)
#2	(((((Depression) OR (Depressive Symptoms)) OR (Depressive Symptom)) OR (Symptom, Depressive)) OR (Emotional Depression)) OR (Depression, Emotional)
#3	(((((((((((((((((((((((((((((((((((((Gastrointestinal Microbiome) OR (Gastrointestinal Microbiomes)) OR (Microbiome, Gastrointestinal)) OR (Gut Microbiome)) OR (Gut Microbiomes)) OR (Microbiome, Gut)) OR (Gut Microflora)) OR (Microflora, Gut)) OR (Gut Microbiota)) OR (Gut Microbiotas)) OR (Microbiota, Gut)) OR (Gastrointestinal Flora)) OR (Flora, Gastrointestinal)) OR (Gut Flora)) OR (Flora, Gut)) OR (Gastrointestinal Microbiota)) OR (Gastrointestinal Microbiotas)) OR (Microbiota, Gastrointestinal)) OR (Gastrointestinal Microbial Community)) OR (Gastrointestinal Microbial Communities)) OR (Microbial Community, Gastrointestinal)) OR (Gastrointestinal Microflora)) OR (Microflora, Gastrointestinal)) OR (Gastric Microbiome)) OR (Gastric Microbiomes)) OR (Microbiome, Gastric)) OR (Intestinal Microbiome)) OR (Intestinal Microbiomes)) OR (Microbiome, Intestinal)) OR (Intestinal Microbiotas)) OR (Microbiota, Intestinal)) OR (Intestinal Microflora)) OR (Microflora, Intestinal)) OR (Intestinal Flora)) OR (Flora, Intestinal)) OR (Enteric Bacteria)) OR (Bacteria, Enteric)) OR

	(Intestinal Microbiota)
#4	#1 AND #2 AND #3

Supplementary table 2-1. Quality of Cohort studies included in the meta-analysis (NOS)

First Author	Year	Selection	Comparability	Outcome	Total
Shanshan Yao	2023	★★★★	★★	★★★	9
Yingjia Wang	2023	★★★★	★★	★★★	9

Supplementary table 2-2. Quality of Cross-sectional studies included in the meta-analysis (AHRQ)

Study	items											Quality score
	①	②	③	④	⑤	⑥	⑦	⑧	⑨	⑩	⑪	
Xuebin Li[2022]	Y	Y	Y	Y	Y	Y	Y	Y	UC	Y	UC	9
Cuiping Huang[2022]	Y	Y	Y	Y	Y	Y	Y	UC	UC	UC	UC	7
Xudong Guo[2022]	Y	Y	Y	Y	Y	Y	Y	Y	UC	UC	UC	8
Lihua Qin[2022]	Y	Y	Y	Y	Y	Y	Y	Y	UC	Y	UC	9
Yi Kang[2021]	Y	Y	Y	Y	Y	UC	Y	UC	UC	UC	UC	6
Yinting Huang[2021]	Y	UC	UC	N	Y	UC	Y	Y	UC	Y	UC	5
Guangshun Han[2021]	Y	Y	Y	Y	Y	Y	Y	Y	N	UC	UC	8
Yanhong Li[2021]	Y	Y	Y	Y	Y	UC	Y	Y	UC	UC	UC	7
Yi Ling[2020]	Y	Y	Y	Y	Y	UC	Y	Y	N	N	UC	7
Xinyue Sun[2019]	Y	Y	N	N	Y	Y	Y	Y	UC	UC	UC	6
Xuecan Zuo[2019]	Y	Y	Y	Y	Y	Y	Y	Y	UC	UC	UC	8
Wentao Fan[2016]	Y	Y	N	N	Y	UC	UC	UC	UC	Y	UC	4

①Define the source of information (survey, record review)②List inclusion and exclusion criteria for exposed and unexposed subjects (cases and controls) or refer to previous publications ③Indicate time period used for identifying patients④Indicate whether or not subjects were consecutive if not population-based ⑤Indicate if evaluators of subjective components of the study were masked to other aspects of the status of the participants ⑥ Describe any assessments undertaken for quality assurance purposes (e.g., test/retest of primary outcome measurements)⑦Explain any patient exclusions from analysis ⑧Describe how confounding was assessed and/or controlled ⑨If applicable, explain how missing data were handled in the analysis⑩ Summarize patient response rates and completeness of data collection⑪Clarify what follow-up if any, was expected and the percentage of patients for which incomplete data or follow-up was obtained. Y: Yes; N: No; UC: unclear

Supplementary table 3. Bias of the 14 studies included in this meta-analysis based on RoBANS.

Study	Comparability of the target group	Target group selection	Confounders	Measurement of intervention/exposure	Blinding of assessors	Outcome assessment	Incomplete outcome data	Selective outcome reporting
Shanshan Yao[2023]	Low risk	Low risk	Moderate risk	Low risk	Low risk	Low risk	Low risk	Low risk
Yingjia Wang[2023]	Low risk	Low risk	Low risk	Low risk	Low risk	Low risk	Low risk	Low risk
Xuebin Li[2022]	Low risk	Low risk	Moderate risk	Low risk	Low risk	Low risk	Low risk	Low risk
Cuiping Huang[2022]	Low risk	Low risk	Moderate risk	Low risk	Low risk	Low risk	Low risk	Low risk
Xudong Guo[2022]	Low risk	Low risk	Moderate risk	Low risk	Low risk	Low risk	Low risk	Low risk
Lihua Qin[2022]	Low risk	Low risk	Moderate risk	Low risk	Low risk	Low risk	Low risk	Low risk
Yi Kang[2021]	Low risk	Low risk	Moderate risk	Low risk	Low risk	Low risk	Low risk	Low risk
Yinting Huang[2021]	Low risk	Low risk	Moderate risk	Low risk	Low risk	Low risk	Low risk	Low risk

Guangshun Han2021	Low risk	Low risk	Moderate risk	Low risk	Low risk	Low risk	Low risk	Low risk
Yanhong Li2021	Low risk	Low risk	Moderate risk	Low risk	Low risk	Low risk	Low risk	Low risk
Yi Ling2020	Low risk	Low risk	Moderate risk	Low risk	Low risk	Low risk	Low risk	Low risk
Xinyue Sun2019	Low risk	Low risk	Moderate risk	Low risk	Low risk	Low risk	Low risk	Low risk
Xuecan Zuo2019	Low risk	Low risk	Moderate risk	Low risk	Low risk	Low risk	Low risk	Low risk
Wentao Fan2016	Low risk	Low risk	Moderate risk	Low risk	Low risk	Low risk	Low risk	Low risk

Supplementary table 4. Publication bias assessments.

	Begg and Mazumdar rank correlation		Egger's regression intercept test		Duval and Tweedie's trim and fill	
	**Tau**	**P value**	**Intercept**	**P value**	**Observed Hedges' g**	**Adjusted Hedges' g**
P-Pseudomonadota	-0.500	0.220	-3.632	0.064		
P-Bacteroidota	0.500	0.308	2.419	0.212		
P-Bacillota	-0.100	0.806	-0.274	0.947		
P-Fusobacteriota	0	1.000	5.217	0.688		
F-Rikenellaceae	0	1.000	15.815	0.089		
F-Acidaminococcaceae	0	1.000	15.760	0.067	9.362[6.168，12.557]	3.253[-0.751，7.257]
F-Fusobacteriaceae	0	1.000	17.570	0.359	3.470[1.988，4.952]	2.703[1.034，4.373]
F-Brassicaceae	0.666	0.296	21.277	0.094	4.302[2.156，6.449]	2.400[0.119，4.682]
F-Enterobacteriaceae	0.500	0.308	29.179	0.050	5.180[1.843，8.517]	2.536[-0.702，5.774]
F-Porphyromonadaceae	0	1.000	12.003	0.301		
F-Erysipelotrichaceae	-0.166	0.734	-20.179	0.111		
F-Bacteroidaceae	0.166	0.734	22.386	0.395		
F-Prevotellaceae	0.833	0.089	1.459	0.013		
F-Ruminococcaceae	0.166	0.734	7.248	0.278		

F-Lachnospiraceae	0.166	0.734	0.096	0.920		
G-Bacillus anthracis	0	1.000	18.355	0.240		
G-Escherichia/Shigella	0.166	0.734	-14.462	0.794		
G-Roseburia	0	1.000	-12.826	0.486		
G-Blautia	0.500	0.308	36.430	0.110	3.639[1.227，6.051]	1.732[-0.801，4.265]
G-Lachnospira	0	1.000	-10.475	0.459		
G-Megamonas	0.500	0.308	10.597	0.196	1.509[0.759，2.259]	1.103[0.282，1.925]
G-Prevotella	0.166	0.734	6.777	0.691	-1.691[-2.330，-1.053]	-1.956[-2.647，-1.265]
G-Ruminococcus	-0.500	0.308	-12.476	0.194		
G-F. prausnitzii	0.166	0.734	4.523	0.900	2.224[-0.201，4.650]	1.444[-0.892，3.782]
G-Curtobacterium	-0.833	0.089	-13.602	0.151		

Note: P: phylum; F: family; G: genus; the observed and adjusted effects sizes were reported only when missing studies were found and corrected by Duval and Tweedie's trim and fill. The Hedges' g was presented as overall effect size [lower limit, upper limit].

Supplementary table 5. Heterogeneity analysis and main effect analysis statistical results of α diversity after remove a excluding a study.

α diversity	k	Heterogeneity			Random effects model				Hedges'sg
					95%CI		Test of null (2-Tail)		
		Q	P-value	I^2	Lower limit	Upper limit	Z-value	P-value	
PSD VS HC									
Chao1	4	27.100	0.000	88.930	0.928	2.410	4.417	0.000	1.669
ACE	5	32.937	0.000	87.856	0.203	1.386	2.633	0.008	0.795
Shannon	5	126.013	0.000	96.826	-0.218	2.220	1.609	0.108	1.001
Simpson	4	27.770	0.000	89.197	-0.157	1.157	1.493	0.136	0.500
					Fixed effects model				
PSD VS stroke									
Chao1	2	0.403	0.525	0.000	-0.091	0.383	1.209	0.227	0.146
ACE	2	0.718	0.397	0.000	-0.507	0.223	-0.761	0.447	-0.142
Shannon	4	1.313	0.725	0.000	-0.239	0.153	-0.430	0.667	-0.043
Simpson	2	0.663	0.416	0.000	-0.816	-0.087	-2.429	0.015	-0.451

(A)

Chao1：PSD VS HC

Study name	Hedges's g	Lower limit	Upper limit	p-Value
Yingjia Wang2023	0.803	0.232	1.375	0.006
Xudong Guo2022	2.590	2.135	3.044	0.000
Xinyue Sun2019	-1.553	-2.321	-0.785	0.000
Xuecan Zuo2019	1.933	1.508	2.358	0.000
Wentao Fan2016	1.286	0.745	1.827	0.000
	1.042	-0.100	2.184	0.074

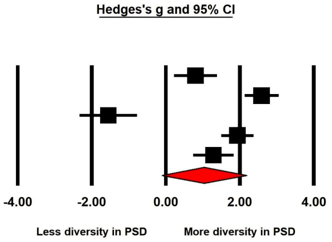

(B)

ACE：PSD VS HC

Study name	Hedges's g	Lower limit	Upper limit	p-Value
Yingjia Wang2023	1.158	0.563	1.753	0.000
Xudong Guo2022	1.178	0.816	1.540	0.000
Guangshun Han2021	-0.438	-0.963	0.086	0.101
Xinyue Sun2019	-1.102	-1.835	-0.370	0.003
Xuecan Zuo2019	1.311	0.925	1.698	0.000
Wentao Fan2016	0.709	0.201	1.216	0.006
	0.501	-0.189	1.191	0.155

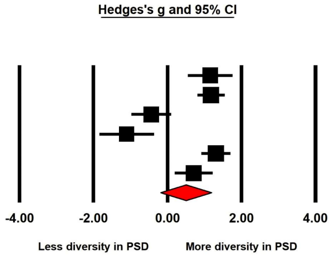

(C)

Shannon：PSD VS HC

Study name	Hedges's g	Lower limit	Upper limit	p-Value
Yingjia Wang2023	0.766	0.196	1.336	0.008
Xudong Guo2022	0.193	-0.142	0.528	0.259
Guangshun Han2021	-0.160	-0.679	0.358	0.545
Xinyue Sun2019	-1.479	-2.241	-0.718	0.000
Xuecan Zuo2019	3.776	3.190	4.362	0.000
Wentao Fan2016	0.473	-0.026	0.972	0.063
	0.601	-0.571	1.773	0.315

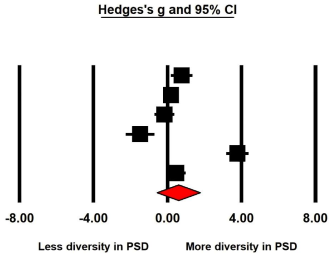

(D)

Simpson：PSD VS HC

Study name	Hedges's g	Lower limit	Upper limit	p-Value
Yingjia Wang2023	0.396	-0.159	0.951	0.162
Xudong Guo2022	0.281	-0.055	0.617	0.101
Xinyue Sun2019	1.457	0.697	2.216	0.000
Xuecan Zuo2019	1.399	1.008	1.790	0.000
Wentao Fan2016	-0.109	-0.602	0.383	0.663
	0.664	0.057	1.271	0.032

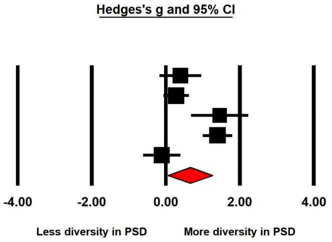

Supplementary Figure 1. Forest plots of Chao1 index (A); ACE indexes (B); Shannon index (C) and Simpson index (D) in the comparisons between post-stroke depression (PSD) and healthy controls (HC) spectrum.
